# Thymol Alleviates LPS-Induced Liver Inflammation and Apoptosis by Inhibiting NLRP3 Inflammasome Activation and the AMPK-mTOR-Autophagy Pathway

**DOI:** 10.3390/nu14142809

**Published:** 2022-07-08

**Authors:** Xiujing Dou, Di Yan, Siqi Liu, Lujia Gao, Anshan Shan

**Affiliations:** Institute of Animal Nutrition, Northeast Agricultural University, Harbin 150030, China; douxiujing@neau.edu.cn (X.D.); yandi163@163.com (D.Y.); lsq15765539460@163.com (S.L.); 15645076250@163.com (L.G.)

**Keywords:** thymol, liver injury, inflammation, NLRP3 inflammasome, mTOR, autophagy

## Abstract

Thymol is a natural antibacterial agent found in the essential oil extracted from thyme, which has been proven to be beneficial in food and medicine. Meanwhile, the NOD-like receptor family pyrin domain-containing 3 (NLRP3) inflammasome and autophagy have been reported to play key roles in the progression of liver injury. However, the effects of thymol on the NLRP3 inflammasome and autophagy in protecting the liver remain unclear. The present study used a mouse model with liver injury induced by lipopolysaccharides (LPS) to investigate the regulatory mechanisms of thymol. We found that thymol alleviated LPS-induced liver structural damage, as judged by reduced inflammatory cell infiltration and improved structure. In addition, elevated levels of the liver damage indicators (alanine transaminase (ALT), aspartate transaminase (AST), and total bilirubin (TBIL)) dropped after thymol administration. The mRNA and protein expression of inflammatory cytokines (tumor necrosis factor (TNF)-α, interleukin (IL)-6, and IL-22), apoptosis-related genes (caspase3 and caspase9), and the activity of apoptosis-related genes (caspase3 and caspase9) were increased in LPS-treated livers, whereas the changes were alleviated after thymol administration. Thymol inhibited LPS-induced increment in lactate dehydrogenase (LDH) activity in primary hepatocytes of the mouse. In addition, thymol protected mice from liver injury by inhibiting NLRP3 inflammasome activation induced by LPS. Mechanistically, the present study indicates that thymol has liver protective activity resulting from the modulation of the AMP-activated protein kinase—mammalian target of rapamycin (AMPK–mTOR) to regulate the autophagy pathway, hence curbing inflammation.

## 1. Introduction

Although functioning crucially in immune response and producing a considerable number of inflammatory mediators, the liver remains susceptible to inflammatory injury [1]. Acute liver failure (ALF) is a severe illness with the characteristics of a significant necrosis of the hepatocytes, rapid deterioration of the hepatic function, and eventually multiple organ failure in patients with no preexisting liver disease [2,3]. On account of the severe morbidity and mortality, finding effective medications to cure ALF and extensively studying the mechanism of ALF are critical.

In addition, some natural products or ingredients have been tested in clinical trials, due to their potential medicinal activity. Many dietary supplements, particularly polyphenols, are reported to be promising candidates for treating various diseases, including inflammations. Traditional Chinese medicine is a key element in ALF therapy [2]. As a natural monoterpene phenolic compound, thymol has the potential to be antimicrobial, anti-oxidative, and anti-inflammatory [4]. Furthermore, thymol is proven to help boost the immune system by improving intestinal barrier function and combating harmful germs [5]. However, the liver-protective impact of thymol under stressful conditions is little known. Understanding the defensive mechanisms seems essential to the practical application of thymol in boosting the body’s defenses.

LPS-induced liver injury is mediated by various pro-inflammatory mediators, including tumor necrosis factor (TNF)-α and interleukin (IL)-6 [6,7]. Apoptosis is also involved in liver injury, leading to characteristic changes including chromosomal DNA fragmentation and caspase activation [8,9,10]. In addition to inflammation and apoptosis, a growing body of research has discovered that the NOD-like receptor family pyrin domain-containing 3 (NLRP3) inflammasome activation directly affects several acute and chronic liver disorders [11]. According to studies, the NLRP3 inflammasome is essential for pro-IL-1β conversion into physiologically active IL-1β, as well as the IL-18 release, the onset of inflammation, and the development of immunological responses [12,13]. Inhibiting the activation of the NLRP3 inflammasome seems to be a useful technique for relieving the inflammation of liver injury.

According to recent research, autophagy inhibits the inflammatory responses by serving as a negative feedback mechanism for NLRP3 assembly [14]. It has been suggested that autophagy plays an essential function in maintaining cellular homeostasis by self-digesting proteins with a long half-life or organelles that have been destroyed in response to external stimuli [15,16]. It is also recognized as having a role in modulating normal hepatic function and the etiology of several liver disorders [17]. The LPS modulates autophagy in the liver, indicating the pivotal role of autophagy in moderating hepatic function [18]; likewise, recent studies suggest that the activation of autophagy can alleviate apoptosis and improve functional recovery from liver failure [1]. Since autophagy is triggered by the inhibition of the kinase mammalian target of rapamycin (mTOR), a critical regulator of autophagy [19], it is worth studying whether hepatocyte autophagy protects the liver against injury.

The present study was designed to explore the liver-protective role of thymol. We hypothesized that thymol alleviates the LPS-induced liver inflammation and hepatocyte apoptosis by inhibiting the NLRP3 inflammasome activation and modulating the AMPK–mTOR autophagy-signaling pathway.

## 2. Materials and Methods

### 2.1. Ethics Approval

The study was conducted according to the Laboratory Animal Management Regulations (revised 2016) guidelines of Heilongjiang Province, China. The Institutional Animal Care and Use Committee of Northeast Agricultural University (NEAU-(2011)-9) approved the animal care and treatment procedures.

### 2.2. Chemicals

The thymol (purity > 98.5%), LPS (*Escherichia coli* 055:B5, L2880), and dimethyl sulfoxide (DMSO, D4540) were obtained from Sigma-Aldrich (St. Louis, MO, USA). The mouse monoclonal anti-β-actin (AA128), HRP-labeled goat anti-rabbit IgG (H + L), and HRP-labeled goat anti-mouse IgG (H + L) antibodies (A0208, A0216) were purchased from Beyotime (Shanghai, China). The following antibodies were used in this work: anti-IκB α (4814, Cell Signaling Technology, Danvers, MA, USA), anti-phospho-NF-κB p65 (Ser536, 3033S, Cell Signaling Technology), anti-NLRP3 (AF2155, Beyotime), anti-IL-1β (AF7209, Beyotime), anti-beclin1 (ab231341, Abcam), anti-ATG7 (AA820, Beyotime), anti-LC3B (ab229327, Abcam, Cambridge, UK), anti-phospho-AMPK-α (Thr172, Cell Signaling Technology), anti-phospho-mTOR (Ser2448, D9C2, Cell Signaling Technology), anti-caspase-3 (WL02117, Wanleibio, Shenyang, China), and anti-cleaved caspase-9 (WL01838, Wanleibio).

### 2.3. Animals and Treatment

Thirty-six BALB/c male mice (7–8 weeks, 18–22 g) were procured from Liaoning Changsheng Biotechnology Company (Benxi, China). Throughout the studies, the mice were kept in a temperature (22 ± 2 °C) and humidity (55 ± 5%) controlled environment with a 12-h light/dark cycle and had free access to commercial standardized basal meals and water. Following an acclimatization period of 5 days, the mice were randomly divided into three groups (*n* = 12/group): Con, LPS, and LPS + Thy. The mice in the Thy group were administered 80 mg/kg body weight (BW) thymol by gavage, while the others were administrated with sterile saline by gavage. The thymol was first dissolved in DMSO, heated to 60 °C at a concentration of 8 g/mL, and diluted in distilled water to 8 mg/mL. Then the volume of the thymol solution was calculated according to the BW of each mouse. On the 34th day, the mice in the control group were given sterile saline intraperitoneally, while the others were given 10 mg/kg LPS intraperitoneally. After 4 h of LPS treatment, the mice were anesthetized by diethyl ether inhalation and killed by cervical dislocation. Blood was drawn by removing the eyeball of each mouse and each blood sample was incubated for 30 min at room temperature before being centrifuged at 3000 rpm for 15 min at 4 °C. The serum’s top layer was kept at −20 °C. The liver tissue was removed and fixed with 4% paraformaldehyde, while the rest was frozen in liquid nitrogen and stored at −80 °C.

### 2.4. Histopathological Analysis

The liver tissues were embedded in paraffin and sectioned at a thickness of 5 μm after being fixed in 4% paraformaldehyde at 4 °C. The sections were stained with hematoxylin and eosin (H&E). An optical microscope was used to capture the images. The severity of liver damage was determined using a five-point scale (0–4 equaling none, slight, mild, moderate, and severe, respectively).

### 2.5. Serum Biochemical Analysis

The alanine transaminase (ALT), aspartate transaminase (AST), total bilirubin (TBIL), albumin (ALB), and total protein (TP) were analyzed with an automatic biochemical analyzer (Roche Cobus Mira Plus, Switzerland) at the Heilongjiang Electric Power Hospital (Harbin, China).

### 2.6. Immunohistochemistry (IHC) Staining

The tests were carried out according to the manufacturer’s instructions (Servicebio, Wuhan, China). The paraffin-embedded liver slides were dewaxed with xylene and rehydrated with graded alcohols. The tissue slices were pressure-cooked in 0.01 M citrate buffer (pH 6.0) for 20 min to extract antigen, then incubated with 3% hydrogen peroxide at room temperature for 25 min to inhibit endogenous peroxidase. Then they were blocked with 3% BSA for 30 min, followed by incubation with indicated primary antibody at 4 °C overnight, and then with HRP-conjugated secondary antibodies for 50 min at room temperature. Finally, the sections were counterstained with hematoxylin after being covered with diaminobenzidine (DAB) color-developing solution and observed with a microscope. The nucleus of hematoxylin stained was blue, and the positive expression of DAB was brownish-yellow.

### 2.7. Quantitative Reverse-Transcription Polymerase Chain Reaction (qRT-PCR)

The total RNA was extracted from homogenous liver tissue using the RNAiso Plus reagent (9109, Takara Bio, Inc., Otsu-Shiga, Japan) and the cDNA was synthesized, according to the manufacturer’s procedure, using the PrimeScript RT Master Mix Kit (RR047A, Takara Bio, Inc.). The qRT-PCR reaction was performed using SYBR Green mix (RR420A, Takara Bio, Inc.). The PCR primer sequences were synthesized by Sangon Biotech Co., Ltd. (Shanghai, China) and are listed in Table 1. The relative mRNA expression levels were calculated using the 2^−ΔΔCt^ method.

### 2.8. Elisa Assay

The Elisa kits for murine TNF-α (JM-02415M2), IL-6 (JM-02446M2), IL-22 (JM-02424M2), IL-18 (JM-02452M2), and IL-1β (JM-02323M2) were purchased from Jingmei (Jiangsu Jingmei Biological Technology Co., Ltd., Jiangsu, China), and performed according to the manufacturer’s guidance.

### 2.9. Western Blotting Analysis

The total protein from the liver tissues (0.1 g) was extracted using a RIPA buffer mix containing 1% PMSF, and the protein lysates were denatured at 95 °C for 10 min. Following the concentration assessed by the BCA Protein Assay kit (P0010, Beyotime), equal proteins per sample were separated on 8–12% SDS-PAGE gels and subsequently transferred onto poly-(vinylidene fluoride) (PVDF) membranes (Millipor, Billerica, MA, USA). The membranes were blocked with 5% nonfat milk for 2 h, followed by immunoblotting with the indicated primary antibodies at 4 °C overnight. It was then incubated with HRP-conjugated secondary antibodies for 1 h at room temperature, followed by signal detection using ECL Substrate (P0018AS, Beyotime). The band’s intensity was quantified using ImageJ software 1.8.0. (National institutes of health, Bethesda, MD, USA), and β-actin was used to normalize the relative intensity of target proteins.

### 2.10. TUNEL Staining

The tests were carried out, according to the manufacturer’s instructions (Servicebio, Wuhan, China). The paraffin-embedded liver tissue of each group was incubated in two changes of xylene for 20 min each and dehydrated in two changes of pure ethanol for 10 min each, followed by dehydrating in gradient ethanol of 95%, 90%, 80%, and 70% ethanol, respectively, for 5 min. Then they were repaired with protease K at 37 °C for 25 min and then covered in permeabilized working solution (0.1% triton) and incubated at room temperature for 20 min. The buffer is incubated at room temperature for 10 min. The TDT enzyme, dUTP and the buffer in the TUNEL kit were mixed at a ratio of 1:5:50. Afterward, the mixture was added to the objective tissue in a flat wet box and incubated at 37 °C for 2 h. Again, it was incubated with the DAPI solution at room temperature for 10 min. Finally, the dyeing sections were observed and photographed using a fluorescence microscope; the nucleus was labeled blue by DAPI, and the positive apoptosis cells were green.

### 2.11. Detection of Caspase3 and Caspase9 Activity

The liver’s relative caspase3 and caspase9 activities were performed using the activity assay kits (C1116, C1158, Beyotime, China), according to the manufacturer’s instructions. The liver homogenate was incubated on ice-cold lysis buffer for 5 min, then centrifuged at 10,000× *g* for 10 min. Subsequently, the supernatant liquid was gathered and incubated with Ac-DEVD-pNA (2 mM) or Ac-LEHD-pNA (2 mM) at 37 °C for 2 h. After incubation, absorbance was measured at 405 nm using a microplate reader (Tecan, Zürich, Switzerland).

### 2.12. Isolation and Culture of Primary Hepatocytes

As previously described, the primary hepatocytes were isolated from the BALB/c male mice (7–8 weeks) [20]. The isolated hepatocytes were cultivated in a humidified atmosphere with 5% CO_2_ at 37 °C. The viability of the cells was assessed using the trypan blue exclusion test.

### 2.13. Detection of LDH Activity

The primary hepatocytes were seeded in 12-well plates in triplicate. For the detection of LDH activity, the cell supernatant was collected after treatment with 120 μM thymol for 20 h, followed by treatment with 500 ng/mL LPS for 4 h. The LDH activity was quantified using the activity assay kit (A020-2, Nanjing Jiancheng, China), according to the manufacturer’s instructions. The cell supernatant was collected and centrifuged at 12,000× *g* for 15 min. Each sample was incubated with the mechanism buffer and coenzyme I in the kit at 37 °C for 15 min, followed by co-incubation of 2,4-dinitrophenylhydrazine at 37 °C for 15 min. Then, NaOH solution was added before incubating again at room temperature for 5 min, and the absorbance was measured at 450 nm using a microplate reader.

### 2.14. Statistical Analysis

The mean ± standard deviation (SD) was used to express the data in GraphPad Prism version 8.0 (San Diego, CA, USA). One-way ANOVA followed by Tukey’s multiple comparisons were used to conduct statistical analysis among multiple groups in SPSS 23.0 (SPSS Inc., Chicago, IL, USA). The standard for statistical significance was *p* < 0.05.

## 3. Results

### 3.1. Thymol Protects Mice from LPS-Induced Liver Injury

**Figure 1:**
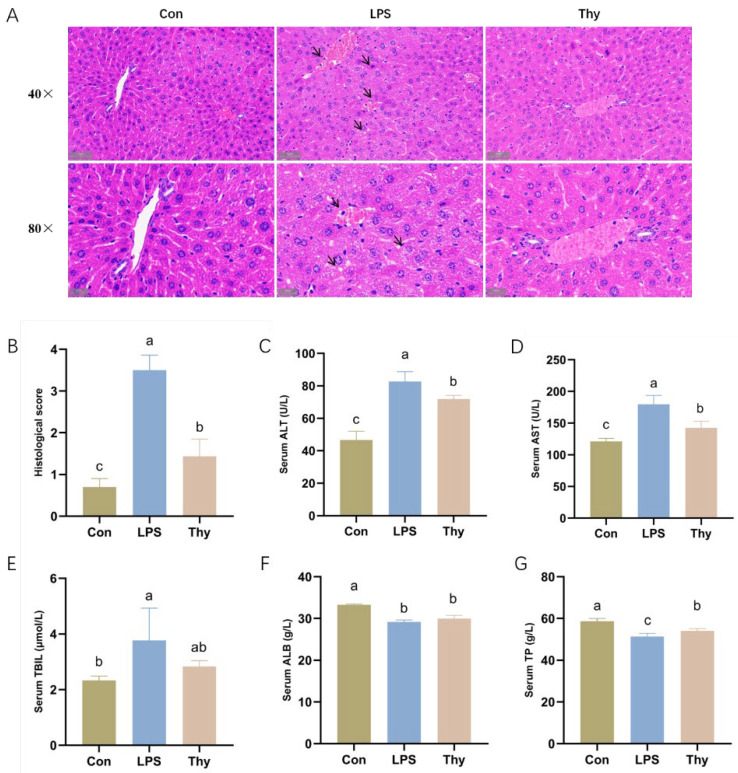
**Thymol administration relieves liver injury induced by LPS.** (**A**) Representative H&E-stained histologic sections of the liver (scale bars: 50 μm, 20 μm); and (**B**) analysis of the histological score. Serum levels of (**C**) ALT; (**D**) AST; (**E**) TBIL; (**F**) ALB; and (**G**) TP (*n* = 3). Con, control; Thy, thymol. Differences were presented with different superscript letters (*p* < 0.05).

No visible liver histopathological changes existed in the Con group. The core veins of the hepatic lobules aligned in a regular pattern, and the cell morphology was normal. On the contrary, apparent histological changes with aberrantly distributed hepatocytes, abnormal cell morphology, inflammatory cell infiltration, and a substantial amount of ruptured hepatic cells were evident after the LPS administration. Compared to the LPS group, the hepatic gross morphology was well maintained with less inflammatory cell infiltration in the Thy group (Figure 1A). When compared consistently to the LPS group, the histological score of the hepatic lesion was significantly decreased upon thymol treatment (Figure 1B).

Furthermore, the activity of the biochemical markers, ALT, AST, and TBIL, increased significantly after the LPS administration. Conversely, the serum ALB and TP levels were obviously lower, indicating that LPS caused liver injury. However, the thymol administration lowered the ALT and AST levels (Figure 1C–G). The above results suggest that thymol has the potential to protect the liver from the LPS challenges.

### 3.2. Thymol Protects Mice from Liver Injury via Suppressing Proinflammatory Cytokines

**Figure 2:**
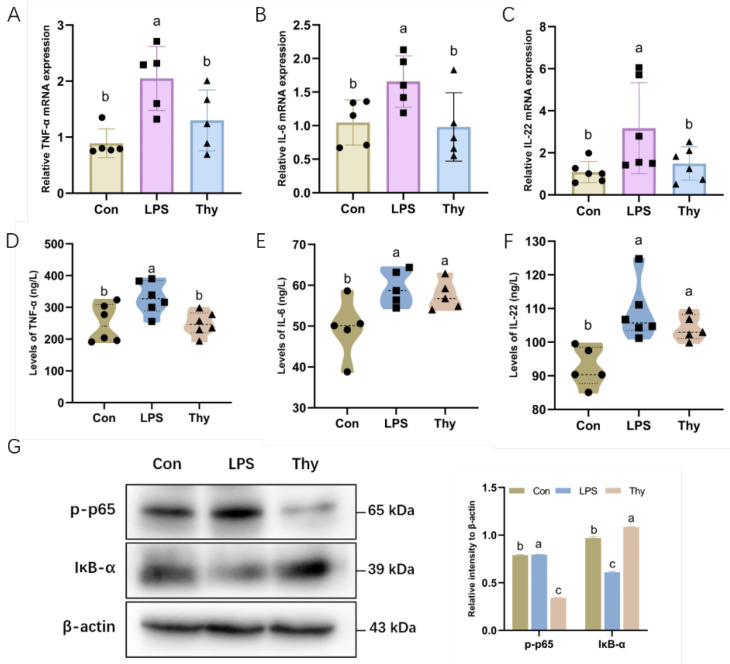
**Thymol ameliorates LPS-induced inflammation.** The mRNA levels of inflammatory cytokines (**A**) TNF-α; (**B**) IL-6; and (**C**) IL-22 in the liver (*n =* 5–6). The concentration of (**D**) TNF-α; (**E**) IL-6; and (**F**) IL-22 in the liver, as determined by ELISA analysis (*n =* 5–6). (**G**) The expression and quantification of p-p65 and IκB-α in the liver as determined by Western blotting. Values are normalized to β-actin levels and expressed as the mean ± SD (*n =* 6). Con, control; Thy, thymol. Differences were presented with different superscript letters (*p* < 0.05).

The overproduction of inflammatory mediators was probably involved in the modulation of the acute-phase response of the hepatic lesions [11]; therefore, the thymol regulation of the inflammatory responses was evaluated to understand its anti-inflammatory properties further. Moreover, controlling the acute-phase response to liver damage and infection may entail the overproduction of inflammatory cytokines [8]. In this study, after the LPS treatment, the mRNA expression of TNF-α, IL-6, and IL-22 increased considerably, indicating that the LPS stimulated the production of these inflammatory mediators (Figure 2A–C), whereas the thymol inhibited the production of the inflammatory mediators, as verified by ELISA (Figure 2D–F). In addition, the thymol mitigated an LPS-induced increase in p65 phosphorylation and a decrease in the IκB-α protein levels (Figure 2G), indicating that thymol plays a role in liver protection via alleviation of the LPS-induced expression of pro-inflammatory cytokines.

### 3.3. Thymol Protects Mice from Liver Injury by Inhibiting NLRP3 Inflammasome Activation

**Figure 3:**
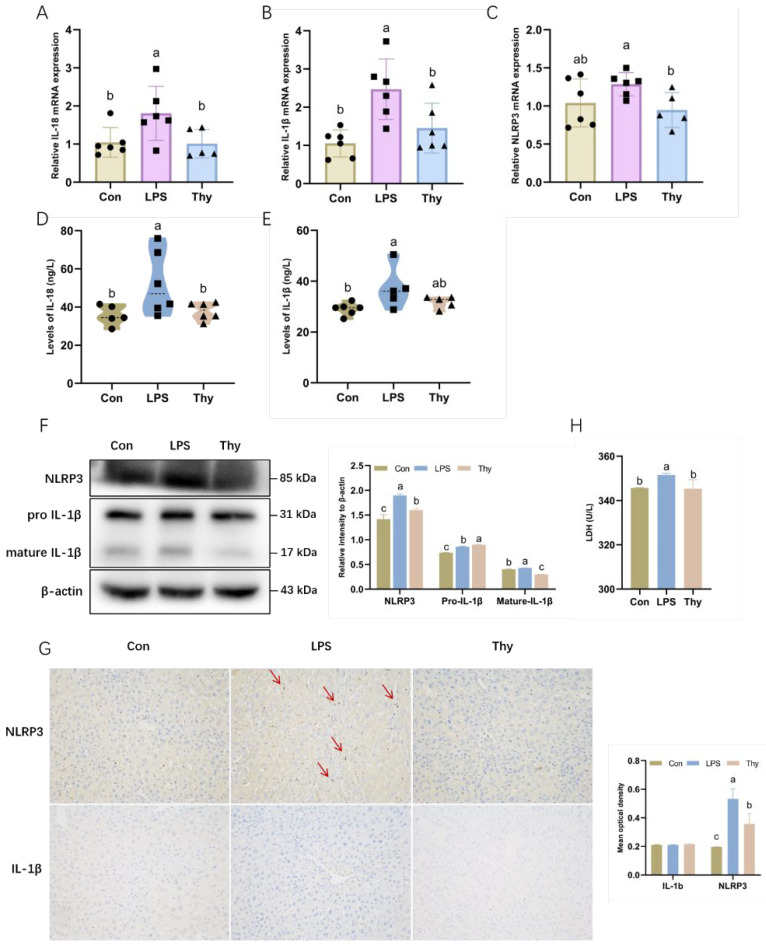
**Tryptophan inhibits the NLRP3 inflammasome to defend against inflammation.** The mRNA expression of (**A**) IL-18; (**B**) IL-1β; and (**C**) NLRP3 in the liver (*n =* 5–6). The concentration of (**D**) IL-18 and (**E**) IL-1β in the liver as examined by ELISA analysis (*n =* 5–6); (**F**) The expression and quantification of NLRP3 and IL-1β in liver as determined by Western blotting. Values are normalized to β-actin levels and expressed as the mean ± SD (*n =* 6); (**G**) Immunohistochemistry of NLRP3 and IL-1β in the liver (200 × magnification). The mean optical density of immunohistochemistry. Values are expressed as the mean ± SD (*n =* 3); (**H**) The activity of LDH in primary hepatocytes (*n =* 3). Con, control; Thy, thymol. Differences were presented with different superscript letters (*p* < 0.05).

In response to caspase1, the inactive precursor forms of pro-IL-1β and pro-IL-18 cleaved into the active form of IL-1β and IL-18 [12]. The assembly and activation of the NLRP3 inflammasome were investigated further based on the suppression of IL-1β and IL-18 by thymol (Figure 3A,B). LPS considerably induced NLRP3 and IL-1β at both mRNA and protein levels, and IL-18 at the protein level (Figure 3C–F). The elevated expressions of NLRP3, IL-1β and IL-18 were dramatically reduced by thymol, which is partially compatible with the immunohistochemistry data (Figure 3G). The activation of the NLRP3 inflammasome was characterized by a significantly increased release of the LDH [21]. In mouse primary hepatocytes, thymol inhibited the LPS-induced increase in LDH activity (Figure 3H). The above results highlight that thymol inhibits the NLRP3 inflammasome activation, which probably helps to explain the decreases in IL-1β and IL-18.

### 3.4. Thymol Protects Mice from Liver Injury via Suppressing Hepatocyte Apoptosis

**Figure 4:**
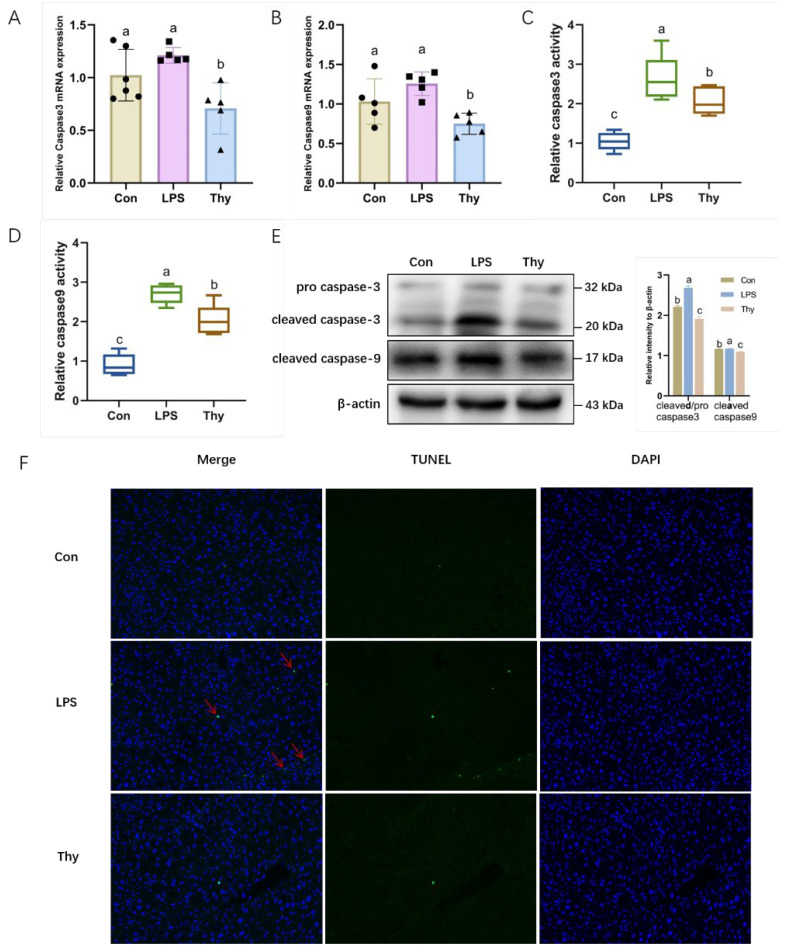
**Thymol ameliorates LPS-induced apoptosis.** The mRNA levels of apoptosis-related genes (**A**) caspase3 and (**B**) caspase9 in liver (*n =* 5–6). The activity of (**C**) caspase3 and (**D**) caspase9 in liver (*n =* 5–6); (**E**) The expression and quantification of pro caspase3, cleaved caspase3, and cleaved caspase9 in liver, as determined by Western blotting. Values are normalized to β-actin levels and expressed as the mean ± SD (*n =* 6); (**F**) TUNEL apoptosis analysis (200× magnification). Con, control; Thy, thymol. Differences were presented with different superscript letters (*p* < 0.05).

As a critical process, the apoptosis of hepatocytes is involved in the early stages of liver injury. Therefore, it was investigated further whether thymol modulated hepatocyte apoptosis to improve liver health. The results showed that, although not statistically significant, the LPS increased the mRNA level of caspase3 and caspase9, which was reversed by thymol (Figure 4A,B). Furthermore, thymol markedly inhibited the LPS-induced increase in the caspase3 and caspase9 activity (Figure 4C,D). The Western blot results consistently showed that the LPS significantly induced the protein expression of the cleaved caspase9 and promoted the conversion of the pro-caspase3 to cleaved-caspase3, which was reversed by thymol (Figure 4E). The TUNEL assay further corroborated these observations, showing that thymol inhibited apoptosis in the LPS-challenged mice (Figure 4F). The above findings suggest that thymol inhibits hepatocyte apoptosis in LPS-induced liver injury.

### 3.5. Thymol Protects Mice from Liver Injury via Modulating the AMPK-mTOR-Autophagy Pathway

**Figure 5:**
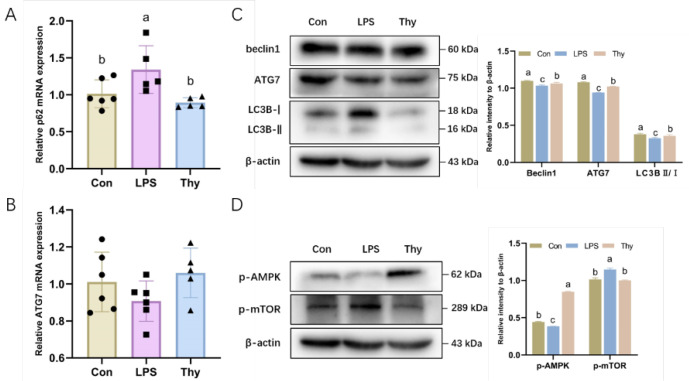
**Thymol regulates the AMPK–mTOR autophagy pathway during inflammatory responses.** The mRNA levels of autophagy-related genes (**A**) p62 and (**B**) ATG7 in the liver (*n =* 5–6); (**C**) The expression and quantification of autophagy-related proteins in the liver as determined by Western blotting. Values are normalized to β-actin levels and expressed as the mean ± SD (*n =* 6); (**D**) The expression and quantification of p-AMPK and p-mTOR in the liver as determined by Western blotting. Values are normalized to β-actin levels and expressed as the mean ± SD (*n =* 6). Con, control; Thy, thymol. Differences were presented with different superscript letters (*p* < 0.05).

The protein expression of the autophagy-associated proteins was examined to clarify the pathways that account for the protective properties of thymol. The transformation from LC3-I to LC3-II is a valid bio-indicator for autophagy activation [17]. Beclin1 is essential in initiating autophagy by being involved in the autophagosomes’ formation [17]. The LPS suppressed the mRNA and protein expression of Beclin1 and ATG7, while it increased p62 in the livers, which was reversed by the thymol pretreatment (Figure 5A–C). The Western blot analysis consistently demonstrated that the LC3-II/LC3-I expression in the liver tissues was decreased by the LPS and alleviated by thymol (Figure 5C).

The kinases mTOR and AMPK are regarded as the indispensable intracellular regulators of autophagy [22]; therefore, the effect of the LPS and thymol exposure on the expression of p-mTOR and its downstream target p-AMPK was determined. Likewise, the impact of the LPS and thymol administration on the phosphorylation of both mTOR and its downstream target AMPK in the liver was investigated in vivo. Interestingly, thymol reversed the LPS-induced mTOR activation and AMPK inhibition (Figure 5D). As mentioned earlier, pretreatment with thymol protects mice against hepatic injury via the modulation of the AMPK–mTOR autophagy pathway.

## 4. Discussion

**Figure 6:**
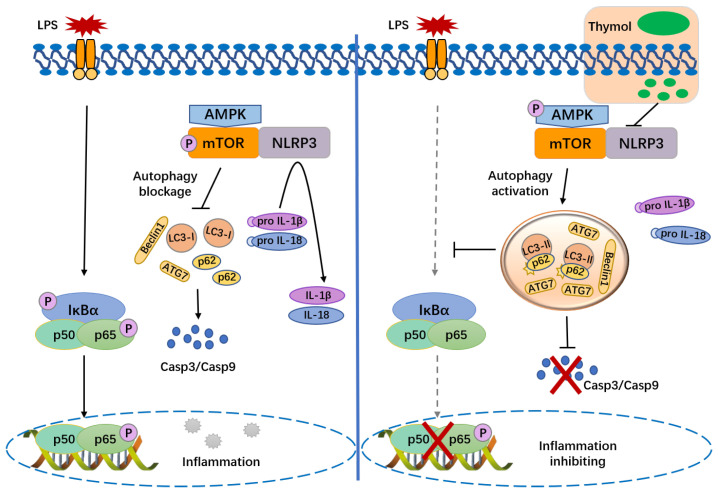
Interaction network of NLRP3 and autophagy after administration of thymol upon LPS challenge.

Due to the involvement of the liver in detoxification and metabolic equilibrium in various ways, liver dysfunction is a widespread concern [22]. The current study demonstrates that thymol pretreatment significantly decreases the liver inflammation and hepatocellular apoptosis, preventing the hepatic injury caused by LPS in mice. Based on these findings, the activation of the NLRP3 inflammasome and modulation of the AMPK–mTOR autophagy-signaling pathway was suppressed by thymol (Figure 6).

When the hepatocytes are injured, the cell membrane integrity is compromised and transaminases (ALT and AST) are released into the blood from hepatocytes, resulting in elevated serum ALT and AST activities [23]. Consequently, ALT and AST are well-known essential biochemical indicators for determining the severity of liver lesions [24]. The content of ALB, a protein generated by the liver that contributes 40–50% of plasma TP, is often associated with the synthesis function of the liver [25]. In this study, LPS-associated hepatotoxicity was indicated by an increase in AST, ALT, and TBIL, and a decrease in TP and ALB, as validated by H&E staining. Thymol could remarkably reduce the content of these markers, which is congruent with the results reported by Yu et al. [26]. Thymol supplementation prevents most, but not all, of the deleterious effects of LPS, which indicates that it has a protective function for the liver lesions.

Hepatic inflammatory responses co-occur with all of the liver diseases and were assumed to be involved in the onset and progression of hepatic lesions [27]. Innate immune cell activation and multiple pro-inflammatory cytokines, including TNF-α and IL-6, trigger and amplify the inflammatory hepatic damage [28]. In the current study, the LPS upregulated the pro-inflammatory mediators TNF-α, IL-6, and IL-22, which were reversed by thymol. The activation of NF-κB, a signaling molecule that can effectively regulate the production of pro-inflammatory proteins, was suppressed by thymol. We hypothesized that thymol could protect the liver from the LPS-induced inflammatory damage. Due to apoptosis or necrosis, chronic and uncontrolled inflammation reportedly causes an enormous loss of hepatocytes and hepatic function [29]. TNF-α, released by active macrophages, has been demonstrated to mediate the hepatocyte injury by binding to the death TNF-α receptor, leading to caspase activation [30]. Furthermore, caspase3 and caspase9 have been implicated in apoptotic reactions on numerous occasions [31]. Compared to the control group, the LPS administration induced the elevated activity of caspase3/9 and the protein expression of cleaved caspase3/9. All of the changes in the hepatic tissue of mice decreased after the treatment with thymol. These observations support the liver-protective role of thymol via anti-inflammation and anti-apoptosis under the LPS challenge.

Recent studies discovered that, apart from triggering inflammatory responses, the LPS stimulation elicited rapid NLRP3 inflammasome activation [32]. The NLRP3 inflammasome is a pattern-recognition receptor in hepatocytes, Kupffer cells, sinusoidal endothelial cells, and hepatic stellate cells, triggered by diverse stimuli including bacteria, viruses, and fungi [33]. The activation of the NLRP3 inflammasome requires two stages: priming and activation [34,35]. NLRP3 recruits ASC and caspase-1 to form the NLRP3 inflammasome, leading to the maturation and secretion of IL-1β and IL-18 [36,37]. It has been proven that the activated NLRP3 inflammasome plays a major role in liver injury. Blocking the NLRP3 inflammasome has been shown to reduce the chronic inflammatory hepatic pathology, which may provide a novel strategy for treating chronic hepatic injury [38,39,40]. Furthermore, accumulated IL-1β and IL-18 play a pro-inflammatory role in hastened liver pathology by recruiting neutrophils [12]. The present study found that thymol significantly reversed the increased expression of genes and proteins related to the assembly (NLRP3) and activation (IL-1β and IL-18) of the NLRP3 inflammasome by LPS in the liver. Additionally, the LPS-induced, increased LDH activity in the primary hepatocytes was inhibited by thymol, implying that thymol was, indeed, involved in the process of hepatocyte injury via NLRP3 silencing.

Increasing studies suggest that autophagy and NLRP3 appear to be regulated by each other. Autophagy is a conserved intracellular mechanism that maintains energy, organelles, and protein homeostasis via a lysosomal breakdown of the damaged macromolecules or organelles, preventing cell damage and promoting cell survival [14]. The previous study indicated that the macrophage autophagy modulates the liver immunological reactivity after the LPS challenge via modulating the inflammasome activation [18]. Furthermore, the autophagy inhibition promotes apoptosis upregulation that is abnormal and exacerbates the hepatocyte dysfunction [1]. All of the preceding findings support that functional autophagy protects the liver against damage. Autophagy suppression causes the buildup of depolarized mitochondria, and the released chemicals subsequently become endogenous inflammasome activators, demonstrating that the NLRP3 degradation is autophagy-dependent [41,42]. Autophagy promotes NLRP3 inflammasome degradation, reduces caspase-1 activation, and inhibits the maturation and production of IL-1β and IL-18 [43]. Depleting the autophagic proteins, such as LC3B and Beclin-1, triggers the NLRP3 inflammasome activation and IL-1β and IL-18 secretion, validating the negative regulation of the NLRP3 inflammasome activation by autophagy [44]. The present study indicated that thymol dramatically restored the LPS-inhibited autophagy by evaluating the levels of genes and proteins linked to autophagy, suggesting that thymol suppresses the inflammasome via activating autophagy. In addition, the pathways by which thymol regulates autophagy in the liver following exposure to LPS were also investigated. Among the multiple pathways regulating autophagy, the AMPK/mTOR axis is classic and well known [45]. When the AMPK is activated as p-AMPK, mTOR is prevented from activating into p-mTOR, leading to autophagy activation [46]. The increased phosphorylation of mTOR and the decreased phosphorylation of AMPK by LPS were observed and, as expected, thymol reversed the LPS-induced changes. These findings reveal that thymol alleviates LPS-regulated autophagy and modulates the AMPK–mTOR pathway.

The exact involvement of autophagy in hepatic damage is unclear. However, according to prior research, the autophagy increased dose-dependently in the mouse heart at low dosages of the LPS, but gradually declined at higher dosages of 5–15 mg/kg [47]. This indicates that autophagy was activated in moderate injury but suppressed in severe injury. The current findings further suggest that autophagy was inhibited in the livers of the LPS-challenged mice, and that the 10 mg/kg LPS delivered for 4 h could also reflect serious liver injury in the mice. In contrast to our findings, researchers revealed that the LPS-induced liver autophagy results in inflammation during sepsis [48]. The distinctions between genetic manipulation and pharmacological modulation probably account for these inconsistencies [49]. The significance of autophagy in inflammatory disease models deserves further discussion.

In conclusion, the present study shows that thymol regulates the NLRP3 inflammasome–autophagy pathway to protect the liver against LPS-induced damage. Therefore, thymol has the potential to be developed into clinical medicine for treating the hepatic injury. For the practical application of thymol against liver injury, more research on the protective mechanisms of thymol, as well as preclinical trials, are required.

## Figures and Tables

**Table 1:** The sequences of primers for qRT-PCR.

Gene	Primer (5′-3′)	Gene Bank Accession
β-actin	F: GTGCTATGTTGCTCTAGACTTCG	NM_007393.5
	R: ATGCCACAGGATTCCATACC	
TNF-α	F: GCCTCTTCTCATTCCTGCTTGTGG	NM_001278601.1
	R: GTGGTTTGTGAGTGTGAGGGTCTG	
IL-6	F: CTTCTTGGGACTGATGCTGGTGAC	NM_001314054.1
	R: AGGTCTGTTGGGAGTGGTATCCTC	
IL-22	F: TTCCAGCAGCCATACATCGTCAAC	XM_006513865.4
	R: GGTAGCACTGATCCTTAGCACTGAC	
IL-1β	F: TCGCAGCAGCACATCAACAAGAG	XM_006498795.5
	R: AGGTCCACGGGAAAGACACAGG	
IL-18	F: GTTAGGTGGGGAGGGTTTGTGTTC	XM_036154619.1
	R: GCAGCCTCGGGTATTCTGTTATGG	
NLRP3	F: CCTGGTCTGCTGGATTGTGTGC	XM_039085397.1
	R: AGTCGTGGTCTTGGAGGTCTGG	
Caspase3	F: TCTGACTGGAAAGCCGAAACTCTTC	XM_017312543.3
	R: GTCCCACTGTCTGTCTCAATGCC	
Caspase9	F: ATGCTGTGTCAAGTTTGCCTACCC	NM_001355176.1
	R: GCTCCAGAATGCCATCCAAGGTC	
p62(SQSTM1)	F: TGGAGTCGGATAACTGCTCAGGAG	NM_175843.4
	R: AGACTGGAGTTCACCTGTGGATGG	
ATG7	F: GGCACGAACTGACCCAGAAGAAG	XM_036152370.1
	R: GCAGACCAGCAGAGTCACCATTG	

## Data Availability

The data will be available from the corresponding author upon reasonable request.

## Abbreviations

NLRP3, NOD-like receptor family pyrin domain-containing 3; ASC, apoptosis-associated speck-like protein containing a caspase recruitment domain foci; LPS, lipopolysaccharides; ALF, acute liver failure; ALT, alanine transaminase; AST, aspartate transaminase; TBIL, total bilirubin; ALB, serum albumin; TP, total protein; TNF, tumor necrosis factor; IL, interleukin; LC3, microtubule-associated protein 1 light chain 3; ATG7, autophagy-related gene 7; AMPK, AMP-activated protein kinase; mTOR, mammalian target of rapamycin; H&E, hematoxylin and eosin; IHC, immunohistochemistry; ELISA, enzyme-linked immunosorbent assay; TUNEL, TdT-mediated dUTP nick end labeling; LDH, lactate dehydrogenase.
